# Estimating ${\hbox {FLE}}_\mathrm{image}$ distributions of manual fiducial localization in CT images

**DOI:** 10.1007/s11548-016-1389-0

**Published:** 2016-03-30

**Authors:** Zoltan Bardosi, Wolfgang Freysinger

**Affiliations:** Medical University Innsbruck, Innsbruck, Austria

**Keywords:** Navigation, Registration, Virtual CT, FLE

## Abstract

**Purpose:**

The fiducial localization error distribution (FLE) and fiducial configuration govern the application accuracy of point-based registration and drive target registration error (TRE) prediction models. The error of physically localizing patient fiducials (${\hbox {FLE}}_\mathrm{patient}$) is negligible when a registration probe matches the implanted screws with mechanical precision. Reliable trackers provide an unbiased estimate of the positional error (${\hbox {FLE}}_\mathrm{tracker}$) with cheap repetitions. FLE further contains the localization error in the imaging data (${\hbox {FLE}}_\mathrm{image}$), sampling of which in general is expensive and possibly biased. Finding the best techniques for estimating ${\hbox {FLE}}_\mathrm{image}$ is crucial for the applicability of the TRE prediction methods.

**Methods:**

We built a ground-truth (gt)-based unbiased estimator ($\widehat{{\hbox {FLE}}_\mathrm{gt}}$) of ${\hbox {FLE}}_\mathrm{image}$ from the samples collected in a virtual CT dataset in which the true locations of image fiducials are known by definition. Replacing true locations in ${\hbox {FLE}}_\mathrm{gt}$ by the sample mean creates a practical difference-to-mean (dtm)-based estimator ($\widehat{{\hbox {FLE}}_\mathrm{dtm}}$) that is applicable on any dataset. To check the practical validity of the dtm estimator, ten persons manually localized nine fiducials ten times in the virtual CT and the resulting ${\hbox {FLE}}_\mathrm{dtm}$ and ${\hbox {FLE}}_\mathrm{gt}$ distributions were tested for statistical equality with a kernel-based two-sample test using the maximum mean discrepancy (MMD) (Gretton in J Mach Learn Res 13:723–773, 2012) statistics at $\alpha =0.05$.

**Results:**

${\hbox {FLE}}_\mathrm{dtm}$ and ${\hbox {FLE}}_\mathrm{gt}$ were found (for most of the cases) not to be statistically significantly different; conditioning them on persons and/or screws however yielded statistically significant differences much more often.

**Conclusions:**

We conclude that $\widehat{{\hbox {FLE}}_\mathrm{dtm}}$ is the best candidate (within our model) for estimating ${\hbox {FLE}}_\mathrm{image}$ in homogeneous TRE prediction models. The presented approach also allows ground-truth-based numerical validation of ${\hbox {FLE}}_\mathrm{image}$ estimators and (manual/automatic) image fiducial localization methods in phantoms with parameters similar to clinical datasets.

## Introduction

Knowing the accuracy of the navigation system is crucial in image-guided surgery. The target registration error (TRE) [2] is the difference between the target position presented by the navigation system and its “true” one. TRE cannot be measured directly except at known fiducial or anatomical locations. All currently available methods to predict the TRE at an arbitrary location use the fiducial localization error distribution (FLE) and depend on the spatial configuration of fiducials. ${\hbox {FLE}}_\mathrm{image}$ and ${\hbox {FLE}}_\mathrm{patient}$ are defined as the distribution of error vectors between measured fiducial locations and their ground-truth positions in image and patient-space, respectively.

In simplest cases of TRE prediction models, the FLE is assumed to be zero-mean isotropic normal distributed [3] but there are extensions for anisotropy [4, 5] and bias [6]. These methods are usually tested with numerical simulations which inherently fulfill all assumptions on the (input) error distributions. Applying prediction models to real-life experiments, however, crucially depends on the characterization of experimental FLE.

This paper studies the simplest case of skull-mounted fiducial screws, as this is known to be the most precise case for point-based registration. In patient-space, ${\hbox {FLE}}_\mathrm{patient}$ is governed by the error of physical fit between the probe and implanted fiducial screws, and the precision of the tracking device. The first part is zero mean and negligible due to the fit of probe and fiducials with mechanical precision; the latter is (assuming proper calibration) zero-mean normal distributed. The cost of tracker measurements is low, so repeated sampling can reduce the jitter induced error.

In image-space, however, the cost of manual localization is high and samples are not guaranteed to be bias-free localizations. Moreover, without knowing the ground-truth fiducial locations the ${\hbox {FLE}}_\mathrm{image}$ distribution cannot be measured in CT datasets. While it is possible to implicitly estimate the combined FLE from the fiducial registration error (FRE) [7], measuring the direct impact of FLE in image-space needs other methods (e.g., [8] approximate ${\hbox {FLE}}_\mathrm{image}$ using intra-modal CT registrations to compare automatic fiducial detection methods for spherical markers in CT images).

This study presents techniques to directly estimate FLE distributions of the human fiducial localization process in image-space without the use of FRE. In order to directly measure FLE, the exact locations of the fiducials have to be known in a reference dataset. When using a physical CT device to create the dataset, complex phantoms have to be manufactured and positioned inside the CT machine with high precision [9]. Recent developments in virtual CT frameworks [10] provide an alternative by generating realistic (with respect to material properties, imaging artefacts, self-shadows, CT sensitivity, and sensor resolution) virtual CT imagery data from complex virtual phantoms without physical image acquisition.

Such a controlled environment is required for ground-truth measurements to evaluate human and algorithmic performance in image fiducial localization. The acquired ground-truth-based measurements are the best estimates that one can ever get for ${\hbox {FLE}}_\mathrm{image}$; therefore, they serve as a reference for evaluating practical estimation methods where ground-truth data are no longer needed. One such estimation method will be presented in the next section.

## Methods

Section “Ground-truth FLE” defines a probabilistic viewpoint on measurement processes and ground-truth-based FLE. Sampling strategies for crowd and single-person-based experimental FLE with and without fiducial orientation dependence are defined. Section “FLE estimation without ground-truth data” defines the “difference-to-mean” (dtm) estimator which does not use the ground-truth data. Section “Testing equality of the gt and dtm estimators” utilizes a distribution-free kernel-based two-sample hypothesis test [1] to check for significant statistical differences between different ground-truth-based reference estimators and their dtm counterparts. The specific measurement process for the experiment is defined in sections “Virtual phantom” and “Data collection”, where the generation of the phantom (virtual CT dataset) and data collection are explained.

### Ground-truth FLE

In this section, we define various alternative interpretations of the FLE distribution, when ground-truth fiducial locations are available. These estimators are assumed to be the best possible estimators of the underlying fiducial localization error distribution. Several parameters (CT resolution, imaging energy levels, postprocessing and reconstruction filters, the fiducial material, size and geometry, etc.) determine the final information content of the dataset in which the localization is made. Other parameters (such as the number of repeated localizations, the fiducial markup software used, the screen resolution) are specific to the procedure with which the data collection is executed. All constant parameters of the imaging process and the measurement methodology are assumed to be implicitly encapsulated in a measurement process $\mathcal {M}$ (e.g., the aforementioned imaging energy levels, postprocessing or reconstruction filters, the resolution and the fiducial materials and sizes, etc., are all process-specific parameters not directly modeled. They are treated as being constants in our investigation). The only explicit parameters of $\mathcal {M}$ modeled are the fiducial set (number, location and orientation of fiducials) and the persons performing the measurements. The following variants of the ground-truth FLE measurement methodologies were differentiated:

In the generic case, a sample $f \in \mathbb {R}^3$ is generated by a measurement process $\mathcal {M}$ with a randomly chosen person *p* on a randomly chosen fiducial *s* at repetition *r*. The values of *p* and *s* are running over all possible persons and fiducials, respectively: $f = \mathcal {M}\left( p, s, r \right)$.

The probability of sampling *f* from $\mathcal {M}$ with uniform selection of *s* and *p* is assumed to follow the probability density function $P_{\mathcal {M}}$

1 $$\begin{aligned} f \sim P_{\mathcal {M}}(\cdot \vert p,s). \end{aligned}$$

Given the true position of fiducials $\mathcal {G}_\mathcal {M} = \left\{ g_1, \dots , g_n \right\} , g_i \in \mathbb {R}^3$ used in $\mathcal {M}$, the ground-truth FLE estimator a 3D vector-valued function of a sample *f* for fiducial $k \in \left\{ 1 \dots n \right\}$ is defined by

2 $$\begin{aligned} \widehat{{\hbox {FLE}}_{\mathrm{gt},k}}(f) := f - g_k. \end{aligned}$$

(2) maps the 3D point sample *f* to the error vector pointing from the true position of fiducial *k* to the acquired sample *f*. The probability distribution (1) induces a probability distribution on the ground-truth FLE vectors as well: $\widehat{{\hbox {FLE}}_{\mathrm{gt},\cdot }(\cdot )}$ over the samples *f* coming from the “$P_{\mathcal {M}}$ conditioned on fiducial k” distribution:

$$\begin{aligned} P_{{\hbox {FLE}}_{\mathrm{gt},k}} = P \left( \widehat{{\hbox {FLE}}_{\mathrm{gt},k}}(f) \vert f \sim P_{\mathcal {M}}(\cdot \vert p, s = k) \right) . \end{aligned}$$

$P_{{\hbox {FLE}}_{\mathrm{gt},k}}$ is therefore the distribution of the errors that occur when the person is randomly chosen for each sample measuring the same fiducial, i.e., the conditional probability of the error vector given the fiducial k. For any given *k*${\hbox {FLE}}_{\mathrm{gt},k}$ defines a distribution of (relative) error vectors; therefore, conditioning on *s* can be interpreted as conditioning on a specific fiducial orientation; to ensure that this holds the test datasets defined all fiducials with a unique orientation. Therefore, $P_{{\hbox {FLE}}_{\mathrm{gt},k}}$ is the orientation-dependent version of ground-truth FLE distribution. Assuming that the samples contain enough different orientations, marginalizing over k gives the fiducial orientation-independent ground-truth FLE distribution

3 $$\begin{aligned} P_{\mathrm{FLE}_\mathrm{gt}} \approx \frac{1}{n} \sum _{k = 1}^{n} P_{\mathrm{FLE}_{\mathrm{gt},k}}. \end{aligned}$$

$P_{{\hbox {FLE}}_\mathrm{gt}}$ is the most generic FLE distribution since it depends neither on person nor on orientation. Person-independent formulations will be referred to as “crowd-based” as they need samples from several individuals.

Since it is only possible to have a finite number of samples from the underlying $P_{{\hbox {FLE}}_\mathrm{gt}}$ distribution, it is impossible to exactly determine it. It is possible however to approximate it with measurements by repeatedly localizing all fiducials with a multitude of participants in a test set containing multiple fiducials with different orientations.

Conditioning $P_{{\hbox {FLE}}_{\mathrm{gt},k}}$ on a person *p* leads to an orientation-dependent and person-specific FLE estimator ($P_{\mathrm{FLE}_{\mathrm{gt},k,p}}$). Conditioning (3) on person *p* results in the orientation-independent person-specific FLE, $P_{{\hbox {FLE}}_{\mathrm{gt},p}}$. The estimated distributions resulting from these estimators are the best possible estimations of the underlying error distributions that we can achieve with finite sampling; therefore, they will be used as reference estimations of ${\hbox {FLE}}_\mathrm{image}$.

### FLE estimation without ground-truth data

This section defines FLE estimation to the practical case when ground-truth fiducial locations are not available in the image dataset. This is the typical case for clinical datasets. The simplest approach [11] is to assume that the measurement process has no bias (the statistical expectation $\mathcal {E} \left( {\hbox {FLE}}_\mathrm{gt}(f) \right) = 0$).

From (2) by replacing $g_k$ (the ground-truth knowledge on fiducial k) with the mean of the samples measuring fiducial k ($\overline{f}_k$) the dtm estimate of the FLE error vector is given by

4 $$\begin{aligned} \widehat{{\hbox {FLE}}_{\mathrm{dtm},k}}(f) := f - \overline{f}_{k}. \end{aligned}$$

With the help of (4) the estimators to all FLE distributions of section “Ground-truth FLE” can be constructed simply by replacing $\widehat{{\hbox {FLE}}_{\mathrm{gt},k}}$ with $\widehat{{\hbox {FLE}}_{\mathrm{dtm},k}}$ in the equations. These estimators are practical because they can be used on any real dataset as well as they do not depend on the ground-truth locations.

### Testing equality of the gt and dtm estimators

The dtm estimator is only useful if the estimated distribution closely captures the underlying distribution (i.e., the “real” ${\hbox {FLE}}_\mathrm{image}$ distribution). Since this underlying distribution is unknown, the best we can hope for is that no statistically significant difference can be found between the ground-truth-based reference distribution (which is the best available unbiased estimation of the underlying distribution ${\hbox {FLE}}_\mathrm{image}$ given the samples) and the dtm-based estimation. Since these distributions are unknown and may differ, a distribution-free two-sample test is needed, where no ordinality of the samples is required, and which tests all moments. Such a test is provided by [1] for the equivalency of distributions using the maximum mean discrepancy metric. The MATLAB code for this test is available from the authors at http://people.kyb.tuebingen.mpg.de/arthur/mmd.htm. For the tests, the usual $\alpha = 0.05$ error level was chosen. The dtm estimator is considered unreliable if the test rejects the null hypothesis that ${\hbox {FLE}}_{\mathrm{dtm}(,*)} = {\hbox {FLE}}_{\mathrm{gt}(,*)}$. Although the lack of evidence for rejection does not prove that the two distributions are equal, it is a clear indicator that they are very similar.

**Fig. 1:**
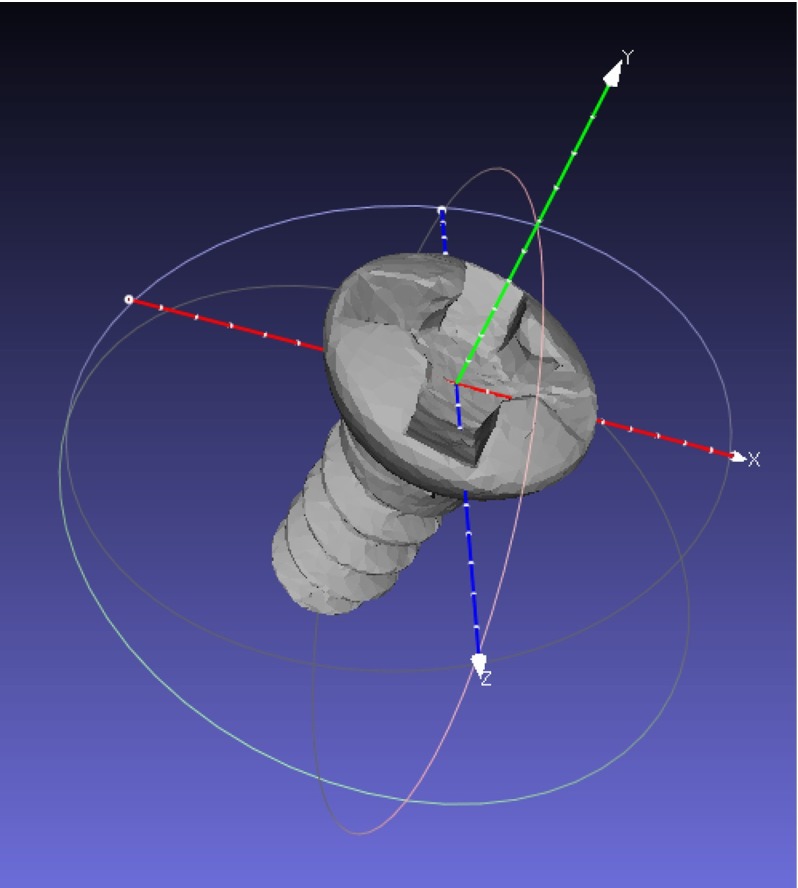
3D mesh of the titanium screw. The origin of the coordinate system is placed at the ground-truth location

**Fig. 2:**
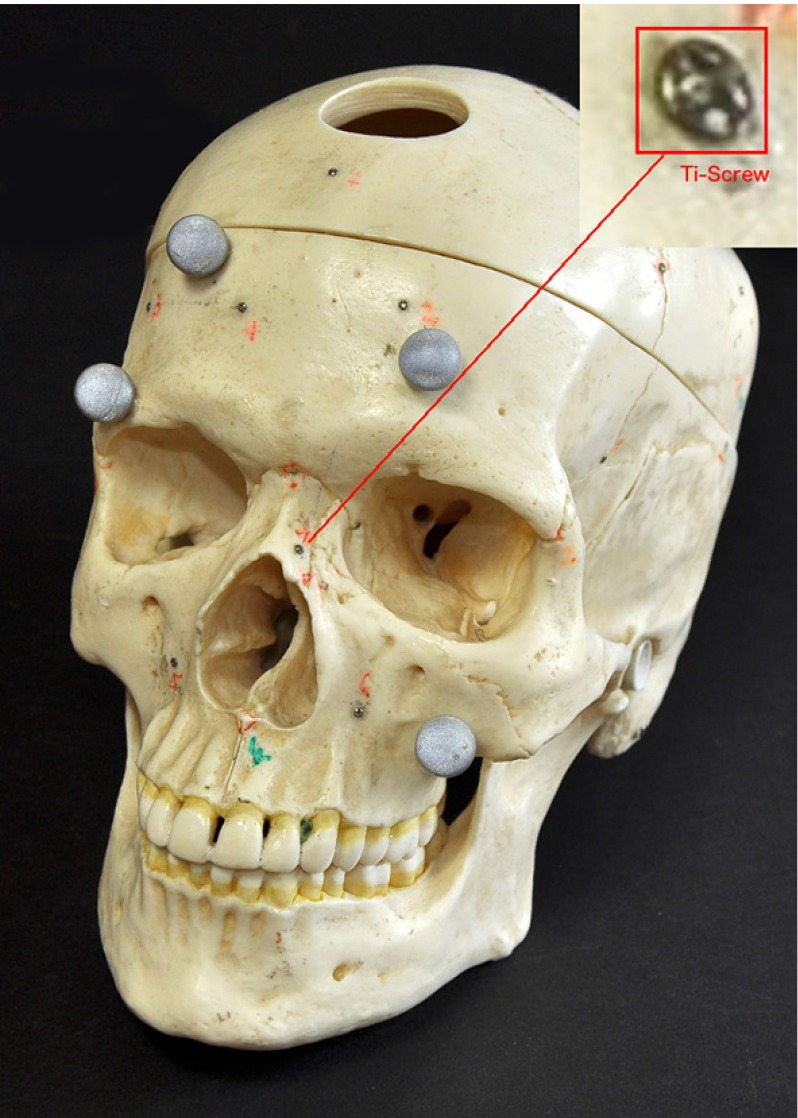
The plastic skull with implanted screw fiducials and attached retroreflective balls for optical tracking. The *top right corner* shows a magnification of an implanted Ti-screw

**Fig. 3:**
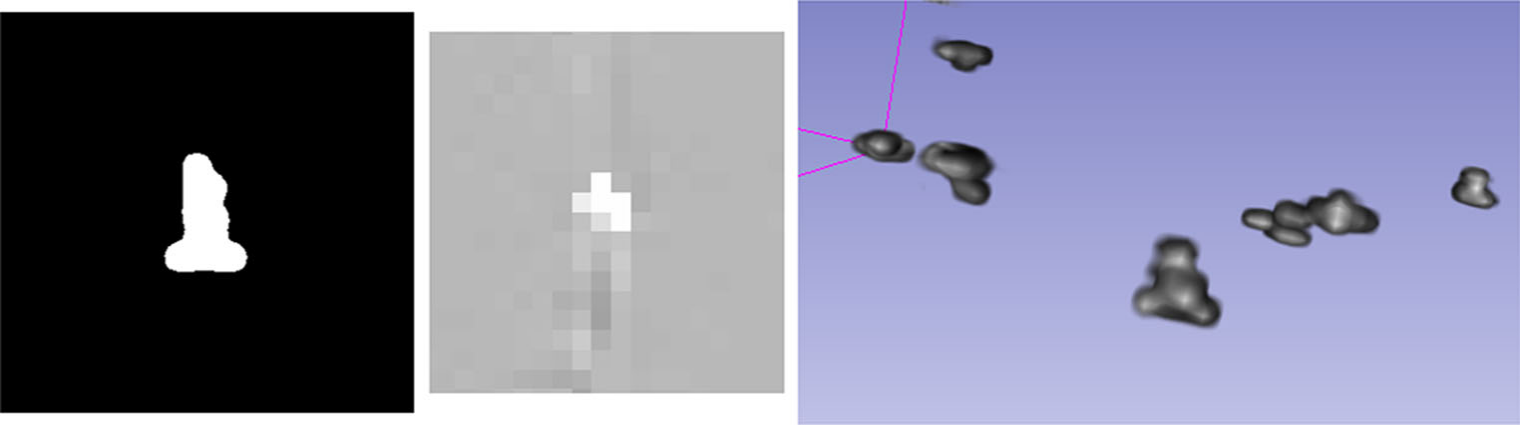
The image on the *left side* shows a cropped sample of the virtual CT image around a screw, after choosing an appropriate Hounsfield intensity window. The *middle image* shows a view of a screw without interpolation and with a different window selection. The image on the *right side* shows a volume-rendered screenshot of the segmented virtual scene within Slicer

### Virtual phantom

In order to collect the required samples to estimate both ground-truth FLE and dtm FLE, a virtual phantom was created. A micro- CT scan of a titanium screw (1 mm $\times$ 3 mm) was used to represent the fiducial geometry. It was scanned in high resolution using a Scanco vivaCT 40 $\mu CT$ (Scanco Medical AG, Switzerland) device at 70 kV, with an image matrix of 2048 $\times$ 2048 pixels and 1000 projections using an isotropic 10.5 $\upmu$m voxel size. The isosurface was thresholded to titanium; the segmentation and mesh generation were done in 3D Slicer [12]. The origin of the mesh was placed at the desired target position where the tracker probe tip is expected to touch the fiducial (Fig. 1). The resulting STL mesh was oriented and positioned into nine different locations in a Blender (www.blender.org) scene. The orientations were randomly chosen but similarly to earlier plastic skull (Fig. 2) phantom experiments [11]. The virtual phantom contained only the virtual screws at these random orientations and positions, and their density was set to match titanium (Fig. 3, right panel).

The CONRAD framework [10] was used to simulate a CT scan of the phantom. The resulting image set was converted to DICOM format (0.4 $\times$ 0.4 $\times$ 1 ${\hbox {mm}}^3$ voxel size) and was imported into 3D Slicer. The screws were annotated and segmented. The resulting scene was used with 3D Slicer throughout the study. The ground-truth positions and orientations of the screws were calculated and saved as reference values. Samples from the virtual CT dataset and the virtual scene are shown in Fig. 3.

**Table 1:** Number of samples used for the different types of estimators

GT Estimator (reference)	dtm Estimator	Number of samples
$P_{{\hbox {FLE}}_\mathrm{gt}}$	$P_{\mathrm{FLE}_\mathrm{dtm}}$	450
$P_{{\hbox {FLE}}_{gt,k}}$	$P_{\mathrm{FLE}_{\mathrm{dtm},k}}$	50 per *k*
$P_{\mathrm{FLE}_{\mathrm{gt},p}}$	$P_{\mathrm{FLE}_{\mathrm{dtm},p}}$	45 per *p*
$P_{\mathrm{FLE}_{\mathrm{gt},k,p}}$	$P_{\mathrm{FLE}_{\mathrm{gt},k,p}}$	5 per (*k*, *p*)

Different sample numbers are due to marginalization and conditioning of the sample set. Also at each iteration only (a randomly subsampled) half of the available samples were used to avoid dependency in the sample pairs

**Fig. 4:**
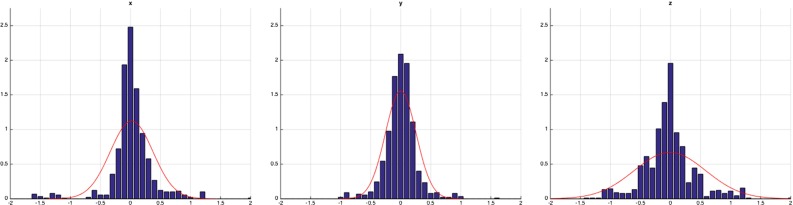
The histogram of ${\hbox {FLE}}_\mathrm{gt}$ vector coordinates in *x*, *y*, *z* (*blue bars*) with the overlay of the maximum likelihood fit Gaussian (*red curve*)

### Data collection

A group of ten individuals (experts and nonexperts) participated in data collection. The experiment consisted of ten repetitions. At each repetition, the participants were asked to localize nine fiducials in the image set. In order to minimize the effect of short-term memory (repeating the same pixel measurement instead of fully reprocessing the dataset), the persons were encouraged to schedule a few hours break between repeated sessions. The participating persons applied the same methodology used on real CT datasets and had no access to the ground-truth locations. Each individual localized each of the 9 screws 10 times, giving 90 samples; in total, 900 samples were obtained.

**Table 2:** The first two moments of the ground-truth crowd-based orientation-independent FLE (${\hbox {FLE}}_\mathrm{gt}$) and its dtm estimator (${\hbox {FLE}}_\mathrm{dtm}$)

Type	$\bar{x}$ (mm)	$\bar{\Sigma } ({\hbox {mm}}^2)$
${\hbox {FLE}}_\mathrm{gt}$	$$ \left( \begin{matrix} 0.0188 \\ 0.0000 \\ -0.0105 \end{matrix} \right) $$	$$ \left( \begin{matrix} 0.1259 &{} 0.0128 &{} 0.0336 \\ 0.0128 &{} 0.0657 &{} -0.0148 \\ 0.0336 &{} -0.0148 &{} 0.3548 \end{matrix} \right) $$
${\hbox {FLE}}_\mathrm{dtm}$	$$ \left( \begin{matrix} 0.0034 \\ 0.0029 \\ 0.0005 \\ \end{matrix} \right) $$	$$ \left( \begin{matrix} 0.1092 &{} 0.0136 &{} 0.0236 \\ 0.0136 &{} 0.0584 &{} -0.0069 \\ 0.0236 &{} -0.0069 &{} 0.3317 \end{matrix} \right) $$

$\bar{x}$, $\bar{\Sigma }$ are sample mean and covariance, respectively

**Fig. 5:**
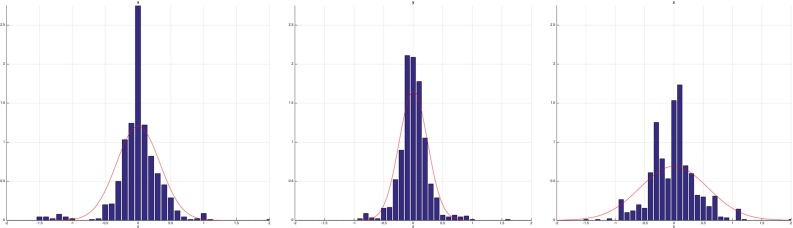
The histogram of ${\hbox {FLE}}_\mathrm{dtm}$ vector coordinates in *x*, *y*, *z* (*blue bars*) with the overlay of the maximum likelihood fit Gaussian (*red curve*)

The definitions of ground-truth and dtm FLE were evaluated, resulting in a set of 3D vectors for each different FLE estimator. Table 1 shows the number of samples used in estimating the various FLE types.

## Results

After data collection, sample means and covariances were estimated for the ground-truth and difference-to-mean estimators using all 900 samples. Figure 4 (and 5) show the histograms of ${\hbox {FLE}}_\mathrm{gt}$ (and FLE$_\mathrm{dtm}$) error coodinates along the CT *x*, *y*, *z* axes and the best fit Gaussian. The collected data are not normally distributed (they fail the Henze-Zirkler, Shapiro-Wilk and Kolmogorov-Smirnov normality tests at $\alpha$ = 0.05 with significant difference to the test threshold), but the Gaussian still visually captures the error spread relatively well. The $\widehat{{{\hbox {FLE}}_\mathrm{dtm}}}$ estimator could only be sampled once as multiple repetitions of the complete experiment were not feasible. Table 2 shows the estimated means and covariances of the crowd-based orientation-independent FLE estimates.

The crowd-based and conditioned versions of the dtm estimators were tested against their ground-truth-based reference at $\alpha = 0.05$ using a null hypothesis threshold determined using bootstrap initialization with 1000 shuffles (as is implemented in the original MMD script). The MMD was used with an RBF (radial basis function) kernel with a kernel width $\sigma = 1.0$. In order not to introduce dependence on the sample pairs used in the hypothesis tests, the original sample set from the experiment was randomly separated into two disjunct sets. The first subset was used to create samples from ${\hbox {FLE}}_{\mathrm{gt}(,*)}$, and the second was used to sample ${\hbox {FLE}}_{\mathrm{dtm}(,*)}$. The random splitting was repeated a 1000 times, and the MMD test was executed on all subset pairs. The rejection rate of the test (which equals to the number of times the null hypothesis was rejected divided by the repetition count) was calculated. The rejection rate can be interpreted as a reliability metric of the estimation as it corresponds to the probability of having a statistically significant difference between the ground-truth reference and the dtm estimate. The lower this value is, the more likely it is to get a practically useful estimate, when applying the estimator. The $\alpha$ level of the applied hypothesis test (which equals the rejection rate when testing a distribution against itself) gives a lower bound on the expected value of the rejection rate. The ${\hbox {FLE}}_{*,p,k}$ estimators were not tested because of the small available sample size (five samples per estimator). Tables 3, 4, and 5 show the estimated rejection rates for all the estimator types.

**Table 3:** Rejection rate for MMD tests $\widehat{{\hbox {FLE}}_\mathrm{gt}}$, $\widehat{{\hbox {FLE}}_\mathrm{dtm}}$

Type	Rejection rate
Crowd	0.1440

**Table 4:** Rejection rates of MMD tests for $\widehat{{\hbox {FLE}}_{\mathrm{gt},p}}$,$\widehat{{\hbox {FLE}}_{\mathrm{dtm},p}}$

*p*	Rejection rate
1	0.562
2	0.592
3	0.161
4	0.128
5	0.163
6	0.236
7	1.0
8	0.367
9	0.438
10	0.173
Mean	0.3820

**Table 5:** Rejection rates of MMD tests for $\widehat{{\hbox {FLE}}_{\mathrm{gt},k}}$,$\widehat{{\hbox {FLE}}_{\mathrm{dtm},k}}$

*k*	Rejection rate
1	0.971
2	0.904
3	0.356
4	0.365
5	0.155
6	0.871
7	0.936
8	0.568
9	0.983
Mean	0.6788

## Discussion and conclusions

In terms of rejection rates, the unconditioned (crowd-based) version of the dtm estimator seems to be the most reliable estimation technique. It seems to describe the data spread; for the crowd case, the zero-mean assumption is also viable. For the majority of the test cases, the resulting estimation shows no statistically significant difference to the ground-truth-based reference distribution.

On the other hand, in the conditioned versions the tests have shown a much higher chance for a person and/or fiducial specific dtm estimate to significantly deviate from the ground-truth reference distribution. After inspection, the primary reason for the difference seems to be systematic bias introduced by the individuals in the markup process. The presence of this bias indicates that in practice more advanced TRE prediction methods are required that are capable of handling the presence of bias in the FLE distributions (e.g., [6]).

Although the advantage of crowd-based data collection is clear, it is questionable if it can become a common practice to determine ${\hbox {FLE}}_\mathrm{image}$ using crowd-based measurements for point-based registrations in image-guided surgery. Crowd-based services (such as Amazon’s MTurk) can potentially be used to estimate ${\hbox {FLE}}_\mathrm{image}$ provided that a sufficient time-span between radiologic patient imaging and surgery is available.

The ground-truth data in the crafted virtual datasets can aid the optimization or evaluation of the performance of persons/algorithms in image fiducial localization by allowing direct numerical validation. Apart from evaluating the performance of other estimators, the ground-truth-based estimators of the presented approach could also provide a sound ${\hbox {FLE}}_\mathrm{image}$ estimate when a tailored virtual CT dataset is used where the imaging parameters are chosen to match the actual clinical values.
